# Studies on experimental animals immunized with different antigenic vaccine combinations of *Neospora caninum* of cattle origin

**DOI:** 10.1186/s13071-025-06687-1

**Published:** 2025-02-10

**Authors:** Zeyu Tang, Zhenyu Wang, Zhen Ma, Weidong Jin, Sicheng Lin, Longsheng Wang, Pengfei Min, Lu Li, Jianhao Zhao, Lijun Jia

**Affiliations:** 1 Engineering Research Center of North-East Cold Region Beef Bovine Science & Technology Innovation, Ministry of Education, Yanbian University, Yanji, Jilin People’s Republic of China; 2 College of Bioengineering, Qingdao University of Science and Technology, Qingdao, Shandong People’s Republic of China

**Keywords:** *Neospora caninum*, GRA9, AMA1, Combined immunization, Mice, Bovine

## Abstract

**Background:**

*Neospora caninum* is an intracellular parasitic protozoon that can infect pregnant animals and cause symptoms such as miscarriage, stillbirth and mummified fetuses. It is one of the main causes of miscarriage in bovines. Apical membrane antigen (AMA) and dense granule protein (GRA) are two major antigenic proteins of *N. caninum.*

**Methods:**

In this study, NcGRA9 recombinant subunit vaccine and Ad5-NcAMA1 recombinant adenovirus vaccine were prepared and used to immunize C57BL/6 mice and Yanbian yellow cattle.

**Results:**

IgG, IgG1, IgG2a, IgG2b, IgA and IgE antibodies and interferon (IFN-γ), interleukin (IL-4) and tumor necrosis factor (TNF-α) cytokines were significantly higher in immunized mice than in the control group (*P* < 0.0001). The biochemical indexes showed that vaccination had no effect on hepatic and renal functions. The survival rate was 70% in mice immunized with NcGRA9 vaccine, 75% in mice immunized with Ad5-NcAMA1 vaccine, 85% in the combined immunization group and 10% in the control group. The parasite load in the brain and liver tissues of the immunized groups was significantly lower than in the control group, as detected by fluorescence quantitative PCR (*P* < 0.0001). In cattle, IgG, IgG2a, IgG2b, IgA, IgM and IgE antibodies and IFN-γ, IL-4 and TNF-α cytokines were significantly higher in the immunized groups than in the control group (*P* < 0.0001). Combined immunization with NcGRA9 + Ad5-NcAMA1 was significantly better than immunization with either vaccine alone.

**Conclusions:**

The biochemical indexes showed that the vaccine had no effect on the liver and kidney functions of cattle. Our results indicate that combined immunization with NcGRA9 + Ad5-NcAMA1 may be a candidate for bovine neosporosis vaccination.

**Graphical Abstract:**

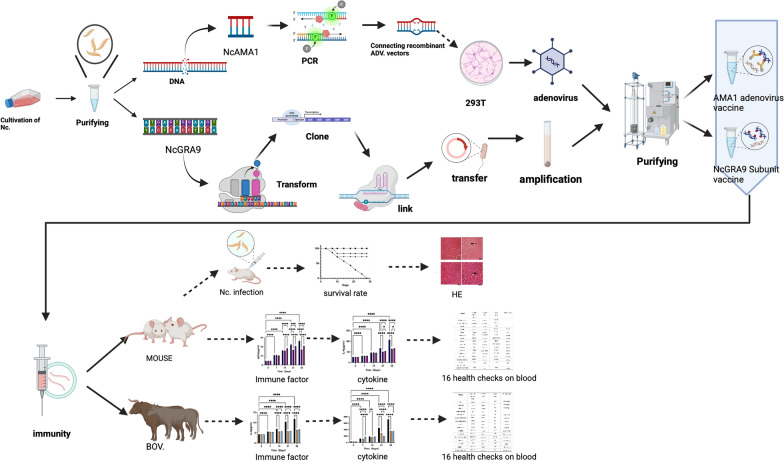

## Background

Neosporosis is a protozoal disease in a wide range of animals. It is caused by *Neospora caninum* (*N. caninum*), which primarily causes abortion, stillbirth or neonatal motor and neurological deficits in female animals [1, 2]. Dogs are the terminal host of *N. caninum*, and intermediate hosts include a variety of mammals. The central nervous system, muscles, liver, brain and other visceral tissues are the main sites of infection by *N. caninum* [3, 4]. Neosporosis has a worldwide distribution, with epidemics occurring in over 30 countries and infection rates ranging from 10 to 40%. With the development of the bovine industry and the frequent trading of bovines, neosporosis has become one of the most important diseases that seriously threaten the healthy development of the industry [5, 6].

Currently, *N. caninum* vaccines include live, inactivated DNA and viral vector vaccines. Live *N. caninum* vaccine has a narrow application range because of its high cost, short storage period, recovery of virulence and ease of pathogen dispersal. Andrianarivo et al. [7] prepared *N. caninum* POYGEN^™^ adjuvanted inactivated vaccine to inoculate pregnant ewes, which induced good humoral and cellular immune responses, but it did not prevent fetal infection. Liddell et al. [8] prepared NcGRA7 and NcsHSP33 DNA vaccines, which induced resistance to *N. caninum* congenital infection in mice. Jenkins et al. [9] used CpG as an adjuvant for NcGRA7 DNA vaccine, and the CpG adjuvant substantially improved the immunoprotection achieved by the DNA vaccine. Nishikawa et al. [10–12] prepared recombinant herpesvirus and recombinant poxvirus vaccines. The recombinant herpesvirus vaccine produced a high level of anti-*N. caninum* IgG antibody in dogs and was able to protect against canine herpesvirus infections. The recombinant poxvirus vaccine induced strong immunoprotection in BALB/c rats and was able to protect against vertical transmission of *N. caninum*.

Candidate molecules for *N. caninum* vaccines have been extensively studied, including surface antigens, glycoprotein-associated sequences of surface antigens, dense granule proteins (GRAs) and apical merozoite antigens (AMAs) [13]. AMA1 is a highly conserved microneme protein in apicomplexan protozoa. AMA1 of *N. caninum* and *Toxoplasma gondii* is a typical single-copy transmembrane protein expressed in bradyzoite and tachyzoite stages [14]. In previous studies, antibodies generated against rNcAMA1 inhibited *N. caninum* and *T. gondii* invasion in mice, but their actual role is not clear [15]. NcGRA9 is present in vesicles as a soluble protein and plays an important role in host cell invasion. Studies have confirmed that the antigenic molecules of GRA2, GRA6 and GRA7 are effective candidate vaccines against *N. caninum*, but little research has been done on NcGRA9 vaccine. Yu et al. [16] inoculated BALB/c mice with a pcNcGRA DNA vaccine constructed from the *N. caninum* GRAs, NcGRA1, NcGRA4, NcGRA9, NcGRA14, NcGRA17 and NcGRA23. These eukaryotic expression plasmids induced production of IgG2a and interferon (IFN-γ), which prolonged survival time and reduced the intracerebral parasite load. This shows the feasibility of NcGRA9 gene as a vaccine antigen. In this study, we prepared NcGRA9 recombinant subunit vaccine and Ad5-NcAMA1 recombinant adenovirus vaccine to evaluate the effect of combined immunization in C57BL/6 mice and Yanbian yellow cattle, aiming to laying a foundation for the prevention and control of neosporosis.

## Methods

### *N. caninum* cultivation and purification

The bovine isolate *N. caninum* was obtained from the brain tissue of bovine fetuses and cultured at the Yanbian University Preventive Veterinary Laboratory, Yanji, China. Vero cells were maintained at the Preventive Veterinary Laboratory of the University. *Neospora caninum* was propagated in Vero cells and cultured in Dulbecco’s modified Eagle's medium (Sigma-Aldrich, St. Louis, MO, USA). The cells were supplemented with 8% heat-inactivated fetal bovine serum (Gibco, Waltham, MA, USA), 100 g/ml penicillin and 10 mg/ml streptomycin (Solarbio, Beijing, China) at 37 °C in a 5% CO_2_ atmosphere. The tachyzoites were purified from the infected Vero cells by washing the cells in ice-cold PBS (PBS; Solarbio). The tachyzoites were filtered through a 50-μm-pore filter to remove the host cell debris and centrifuged at 600 × *g* for 10 min. A hemocytometer was used to count parasites [17, 18].

### Animals

Forty C57BL/6 mice were purchased from Liaoning Changsheng Biotechnology Co., Ltd. (Shenyang, Liaoning Province, China). Twenty adult female Yanbian yellow cattle were obtained from the Antu Beef Breeding Farm (Antu, China). All experimental animals were housed under the same conditions, with sufficient light and appropriate temperature and humidity. The cattle met the following criteria: (i) no other hidden diseases, no infectious diseases and in good physical health and mental state; (ii) complete genetic pedigree and free from hereditary diseases; (iii) no pregnancy for 2 years. All protocols and procedures involving animals were carried out in accordance with the Regulations on the Administration of Laboratory Animal Affairs (Ministry of Science and Technology, China) and approved by the Animal Welfare Committee of Yanbian University, China. During the experimental period, the animals were kept in the same environment and fed the same diet.

### Chemicals and reagents

*BamHI* and *NotI* enzymes, GST purification kit and BCA kit were purchased from Shanghai Sangong Co. HRP-labeled goat anti-mouse IgG was purchased from Abcam (Shanghai, China). ColorMixed Protein Marker, PBST and SDS-PAGE preparation kit were purchased from Beijing Solebaum. Trans5α cloning vector and *Escherichia coli* BL21 were purchased from TransGen Biotech Company (Beijing, China). Mouse anti-*N. caninum* serum was prepared by the Laboratory of Preventive Veterinary Medicine (Yanbian University, Yanji, China). Mouse ELISAs for IgG, IgG1, IgG2a, IgG2b, IgA, IgE, IFN-γ, interleukin (IL-4) and tumor necrosis factor (TNF-α) were purchased from Shanghai Langdon Biotech. ELISAs for bovine IgG, IgG2a, IgG2b, IgA, IgM and IgE as well as mouse cytokine IFN-γ, IL-4 and TNF-α were purchased from Shanghai mlbio Biotech. FastPure Cell/Tissue DNA Isolation Mini Kit, FastPure Gel DNA Extraction Mini Kit, Plasmid Extraction Kit, DL 2000DNA Marker, DL 5000DNA Marker and Ex Taq enzyme were purchased from Nanjing Novozymes Biotechnology Co., Ltd.

### NcGRA9 gene cloning and expression

The genomic DNA of *N. caninum* was extracted using the genomic DNA extraction kit; primers were designed according to the NcGRA9 (NCLIV_066630) gene sequence. The primer sequences were forward: TCTTATCGGGATCCATGCAGGGCGTGAC; reverse: ATAAGAATGCGGCCGCTATTTCCGTTATGGGCGTTCG. Polymerase chain reaction (PCR) amplification of NcGRA9 gene was carried out under the following reaction conditions: pre-denaturation at 94 °C for 5 min, denaturation at 94 °C for 45 s, annealing at 56 °C for 45 s and extension at 72 °C for 45 s, for 35 cycles; final extension at 72 °C for 7 min. The PCR products were recovered by agarose gel electrophoresis and ligated into the pMD18-T Simple Vector to construct a recombinant clone of NcGRA9 gene, which was then subjected to PCR and  catalyst digestion. The correctly identified recombinant plasmid was sent to Shanghai Sangon Biotech. The correctly identified NcGRA9 gene was ligated to the PGEX-4 T-1 vector, and the recombinant plasmid was extracted and transformed into *E. coli* BL21 (DE3), which was then coated on solid LB medium (concentration of LB medium 100 μg/ml). Single colonies were selected and inoculated into liquid LB medium, 100 μg/ml ampicillin was added, and bacteria were cultured at 37 °C and 220 r/min until optical density (OD) reached 0.6–0.8. Isopropyl β-d-1-thiogalactopyranoside (IPTG) was used to induce expression of GRA9 protein at a final concentration of 1 mM for 3 h.The NcGRA9 protein coupled to a glutathione S-transferase (GST) label was induced in *E. coli* (30 °C, 3 h, 200 rpm/min) and purified according to standard steps. The purified protein was recombined with Glutathione Sepharose magnetic beads (GE Healthcare, Waukesha, WI, USA) and then incubated overnight at 4 °C. Finally, the complexes were detected by western blotting and Coomassie brilliant blue staining.

### NcGRA9 protein identification by western blotting

After inducing expression of NcGRA9 protein, organisms were collected and centrifuged at 4000 r/min for 20 min. The supernatant was discarded and the precipitate washed with PBS. Freeze-thaw was carried out three times and Ultrasonic Crush for 20 min to obtain total protein. The total protein was centrifuged at 3000 g for 20 min. We collected the supernatant and added 10 μl of 5 × loading buffer, followed by treatment with boiling water for 10 min. The resulting NcGRA9 protein was identified by western blotting.

### Western blotting

Total protein was extracted using a lysis buffer (Pierce, Rockford, IL, USA) and quantified with the Bradford method. Fifty micrograms of the total protein samples was separated by 10% sodium dodecyl sulfate-polyacrylamide gel electrophoresis (SDS-PAGE) and transferred onto polyvinylidene fluoride membranes (PVDF; Millipore, Billerica, MA, USA). Membranes were incubated overnight at 4 °C with the following primary antibodies. Membranes were washed and subsequently incubated with peroxidase-conjugated anti-mouse or anti-rabbit IgG (Santa Cruz Biotechnology) at 37 °C for 2 h. Bound proteins were visualized using electrochemiluminescence and detected with a bio-imaging system.

### Purification of recombinant proteins and subunit vaccine preparation

The precipitate of *E. coli* was suspended in buffer. After three cycles of freezing and thawing, the bacteria were disrupted by sonication, and the supernatant was collected by centrifugation (400 g, 30 min). The collected protein supernatant was added to a well-equilibrated GST resin purification column. The flow-through solution was run through the purification column again to achieve the highest loading capacity. The GST resin was rinsed by adding 5 × binding buffer. The target protein was eluted with 2 × buffer. The purified NcGRA9 recombinant protein was mixed with Freund's adjuvant to prepare the subunit vaccine.

### Preparation of AMA1 recombinant adenovirus vaccine

Primers were designed using the AMA1 gene sequences of *N. caninum* (AB265823.1), as described previously [14]. After linearization of the plasmid ADV4-NcAMA1 and the framework plasmid pacAd5, Ad5-NcAMA1 recombinant adenovirus was formed after transfection into 293 T cells. The virus titer was determined using the method (BT = PFU/ml). Ad5-NcAMA1 was purified using a Biomiga Virus Purification Kit (San Diego, CA, USA).

### Animal experimentation

Forty mice were randomly divided into four groups of 10: NcGRA9 subunit vaccine group, Ad5-NcAMA1 adenovirus recombinant vaccine group, combined immunization group and control group. The combined immunization group was immunized with NcGRA9 subunit vaccine for the first two immunizations, followed by Ad5-NcAMA1 adenovirus recombinant vaccine (NcGRA9 + Ad5-NcAMA1) for the third and fourth immunizations. The amount of vaccine in each immunization group was 100 µg/vial. The control group was inoculated with an equal amount of PBS. Each immunization group was injected with the vaccine intramuscularly on days 0, 7, 14 and 21. Blood samples were collected via the tail vein before immunization on days 0, 7, 14 and 21 and then on day 28.

### Immunoglobulin subclass and cytokine assays

ELISA was performed to determine the levels of immunoglobulin G (IgG), immunoglobulin G2a (IgG2a), immunoglobulin G2b (IgG2b), immunoglobulin A (IgA), immunoglobulin M (IgM) and immunoglobulin E (IgE) as well as interferon-γ (IFN-γ), interleukin-4 (IL-4) and tumor necrosis factor-alpha (TNF-α) cytokines in the serum of immunized mice. All ELISA kits and antibody information are sourced from Mlbio Shanghai, China. Follow the instructions of the ELISA kit to determine the total protein concentration. In brief, 50 µl of standards and samples was added to the sample wells, followed by 100 µl horseradish peroxidase (HRP)-labeled detection antibody. The contents of the wells were thoroughly mixed by gentle swirling. After a sufficiently long incubation, 100 µl chromogenic substrate was added to each reaction and incubated for 15 min at 37 °C in the dark. The reactions were terminated by adding 50 µl sulfuric acid and swirling gently to mix the contents of the wells. The ELISAs were read at 450 nm, and OD was measured at 50 nm.

### Health 16 check in mice

Blood was collected from mice in an aseptic environment, and 16 hematological indices were measured using a fully automated animal biochemical analyzer (BK-200; Chengdu Smart Technology Co. Ltd., China) to evaluate the effects of the vaccine on liver and kidney functions.

### Immune response to *N. caninum*

At the end of the fourth immunization, 10^6^ *N. caninum* was inoculated intraperitoneally. The vaccine protection test was carried out to observe the clinical symptoms in the mice and determine the survival rate of the mice.

### Quantitative PCR for the detection of changes in *N. caninum*

Quantitative PCR was used to detect the parasite load in brain, heart, liver, spleen, lungs and kidneys of each group of mice. Tissue genomic DNA was extracted using the tissue DNA extraction kit (Tiangen, Beijing, China). The Nc-5 gene (GenBank accession number X842381) was synthesized, as described by Tang [17], and fluorescent quantitative primers were designed and synthesized by Comate Bioscience (Changchun, China). The amplified fragment size was 76 bp and annealing temperature was 60 °C. The primer sequences were as follows: forward: ACTGGAGGCACGCTGAACAC; and reverse: AACAATGCTTCGCAAGAGGAA. Quantitative PCR was performed to detect changes in the quantity of *N. caninum*. The reaction sequence was pre-denaturation at 95 °C for 5 min, denaturation at 95 °C for 10 s and annealing at 60 °C for 30 s. A total of 40 cycles were performed.

### Vaccination of Yanbian yellow cattle

Twenty cattle were randomly divided into four groups of five and immunized using the same schedule as for the mice: NcGRA9 subunit vaccine group, Ad5-NcAMA1 adenovirus recombinant vaccine group, NcGRA9 + Ad5-NcAMA1 combined vaccine group and control group. In the NcGRA9 + Ad5-NcAMA1 group, the first two immunizations were with NcGRA9 subunit vaccine, and the third and fourth immunizations were with Ad5-NcAMA1 adenovirus recombinant vaccine. The vaccine dose was calculated based on the body weight of the cattle, and the amount of vaccine in each group was about 5 ml/head. Vaccine was administered by cervical intramuscular injection, and blood was collected from the jugular vein before each immunization. The grouping of animals, time of immunization and time of blood collection were the same as for the mice.

### Detection of immunoglobulin subclasses and cytokines in serum of cattle

Before each immunization and after the fourth, whole blood was collected, and the serum was separated. ELISAs were applied to detect serum levels of immunoglobulin G (IgG), immunoglobulin G2a (IgG2a), immunoglobulin G2b (IgG2b), immunoglobulin A (IgA), immunoglobulin M (IgM) and immunoglobulin E (IgE) as well as interferon-γ (IFN-γ), interleukin-4 (IL-4) and tumor necrosis factor-alpha (TNF-α).

### Health 16 checks in cattle

Two weeks after the fourth immunization, anticoagulated blood was collected from Yanbian yellow cattle, and a fully automatic animal biochemical analyzer (BK-200; Chengdu Smart Technology) was used to determine the effect of the vaccine on the liver and kidney functions.

### Statistical analysis

All values were expressed as mean ± SD. One-way analysis of variance was used. All statistical analyses were performed using SPSS 20.0 software (Chicago, IL, USA). *P* < 0.05, *P* < 0.001, *P* < 0.0005 and *P* < 0.0001 were considered statistically significant.

## Results

### Cloning and expression of *N. caninum* NcGRA9

**Fig. 1:**
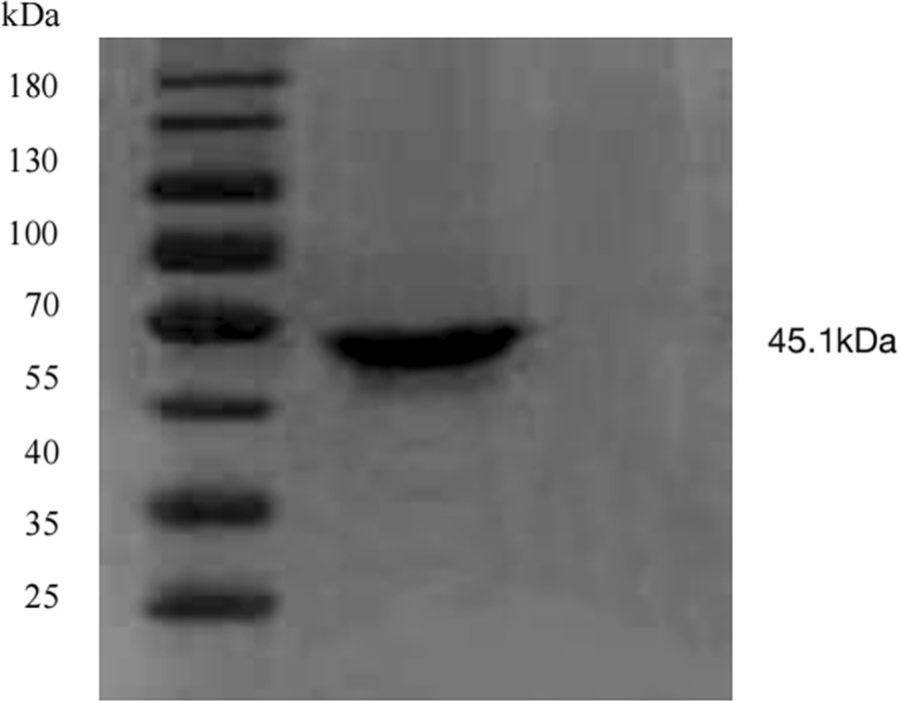
Western blot analysis of recombinant protein. M: Protein marker. 1: Recombinant protein NcGRA 9. 2. Blank control

Using the DNA of *N. caninum* as a template, a 522-bp band was amplified by PCR, and the recombinant plasmids pMD-18 T-NcGRA9 and PGEX-4 T-NcGRA9 were constructed. NcGRA9 had a molecular weight of 45.1 kDa after western blot analysis, and the purified recombinant proteins had good reactivity (Fig. 1).

### Detection of immunoglobulins in the sera of immunized mice

**Fig. 2:**
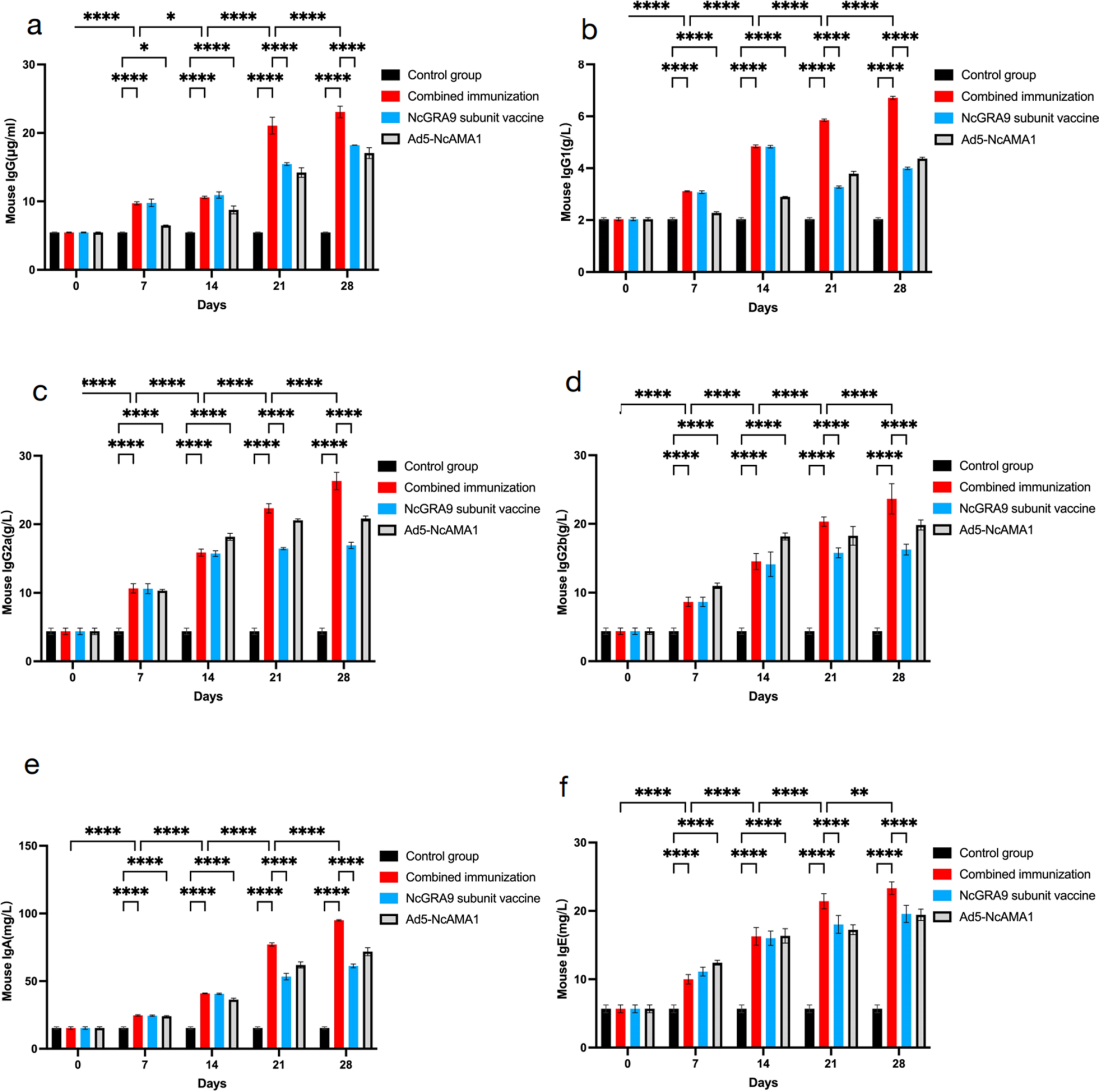
The serum immune antibody levels of mice in different immune groups (combined immune group; NcGRA9 group; Ad5-NcAMA1 group). **a** ELISA showing IgG level of mice; **b** ELISA showing IgG1 level of mice; **c** ELISA showing IgG2a level of mice; **d** ELISA showing IgG2b level of mice; **e** ELISA showing IgA level of mice; **f** ELISA showing IgE level of mice. *P < 0.05, ** *P* < 0.01, *** *P* < 0.001, **** *P* < 0.0001

The levels of IgG, IgG1, IgG2a, IgG2b, IgA and IgE in the sera of immunized mice were detected by ELISA (Fig. 2), which increased significantly with the number of immunizations (*P* < 0.0001). IgG and IgG1 were significantly lower in the Ad5-NcAMA1 group than in the NcGRA9 group on days 7 and 14 of immunization (*P* < 0.0001). On days 21 and 28, the serum levels of IgG1, IgG2a, IgG2b and IgA in the Ad5-NcAMA1 group were significantly higher than those in the NcGRA9 group. In contrast, the levels of IgG were reversed and lower in the Ad5-NcAMA1 group than in the NcGRA9 group (*P* < 0.05 or *P* < 0.001). The levels of IgG and IgE were lower in the Ad5-NcAMA1 group than in the NcGRA9 group (*P* < 0.05 or *P* < 0.001). In the NcGRA9 + Ad5-NcAMA1 group, on days 21 and 28, the serum levels of IgG, IgG1, IgG2a, IgG2b, IgA and IgE showed a highly significant increase (*P* < 0.05 or *P* < 0.0001).

### Detection of serum cytokines in immunized mice

**Fig. 3:**
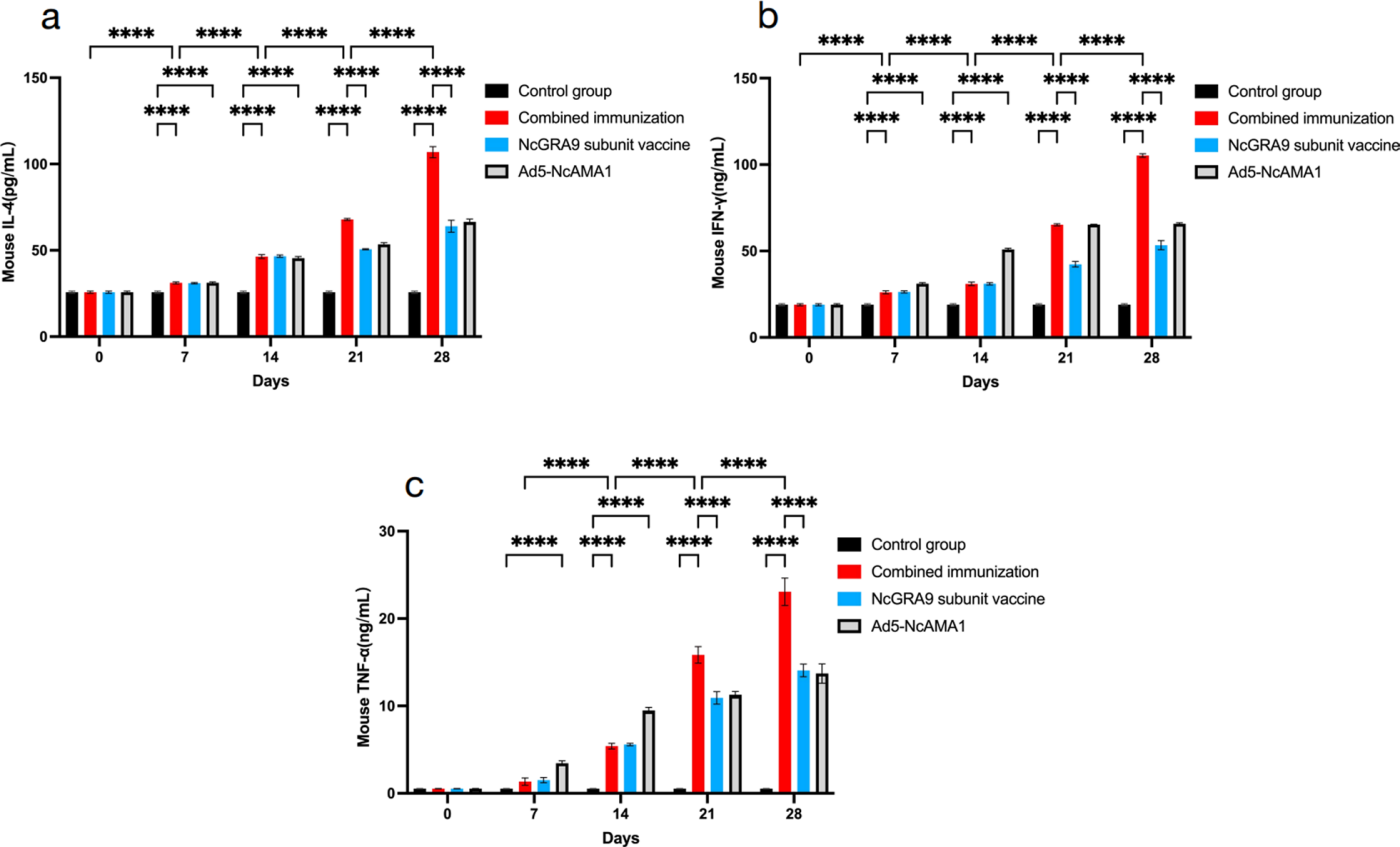
Cytokine antibody levels of mice in different immune groups (combined immune group; NcGRA9 group; Ad5-NcAMA1 group). **a** ELISA showing IL-4 level of mice; **b** ELISA showing IFN-γ level of mice; **c** ELISA showing TNF-α level of mice. *P < 0.05, ** *P* < 0.01, *** *P* < 0.001, **** *P* < 0.0001

The levels of cytokines IFN-γ, TNF-α and IL-4 in the serum of immunized mice were measured using ELISA (Fig. 3). IFN-γ levels in the Ad5-NcAMA1 group were significantly higher than those in the NcGRA9 group from days 7 to 28 (*P* < 0.0001). TNF-α levels in the NcGRA9 group were significantly higher than in the Ad5-NcAMA1 group from days 7 to 28 (*P* < 0.01 or *P* < 0.0001). At days 21 and 28, the levels of IFN-γ, TNF-α and IL-4 in the NcGRA9 + Ad5-NcAMA1 group were significantly higher than those in the groups immunized with either vaccine alone (*P* < 0.0001).

### Sixteen tests for mouse health after immunization

Anticoagulated blood was collected from mice and analyzed by fully automatic animal biochemical analyzer (Table 1). There were lower levels of total bilirubin and creatinine and higher levels of creatine kinase in the immunized mice, but there was no significant difference compared with the normal control group (*P* > 0.05). This indicated that the vaccine had no effect on the liver and kidney functions.

**Table 1:**
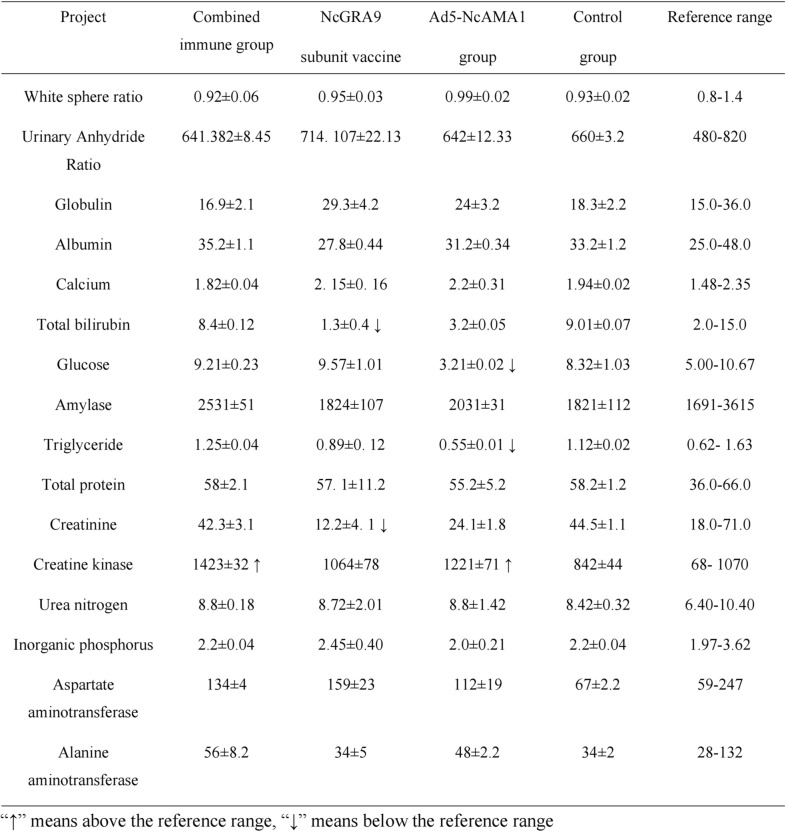
Sixteen health tests of mice after immunization

### Effect of immunization on survival rate of mice infected with *N. caninum*

**Fig. 4:**
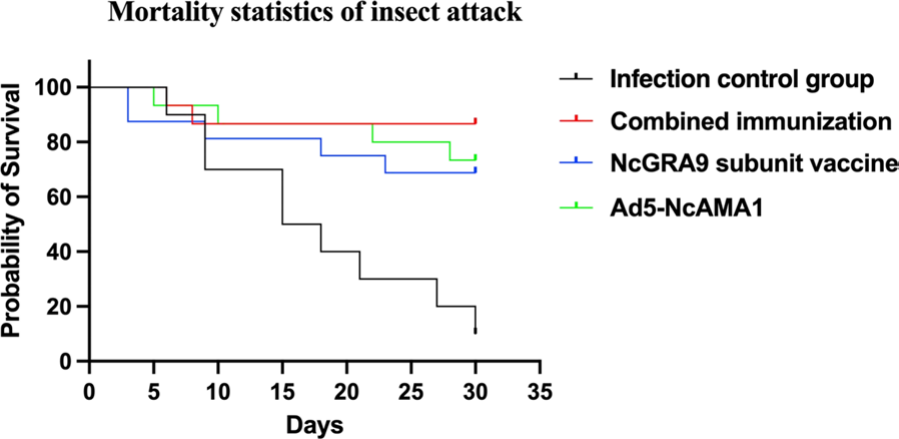
Mortality statistics of insect attack. **Black** infection control group; **red** combined immunization; **blue** NcGRA9 subunit vaccine; **green** Ad5-NcAMA1

The results of the parasite challenge test in C57BL/6 mice are shown in Fig. 4. After challenge with *N. caninum*, the survival rate was 70% in the NcGRA9 group, 75% in the Ad5-NcAMA1 group and 85% in the NcGRA9 + Ad5-NcAMA1 group. The survival rate in the control group was only 10%.

### Effect of immunization on the parasite load in the major organs of mice infected with *N. caninum*

**Fig. 5:**
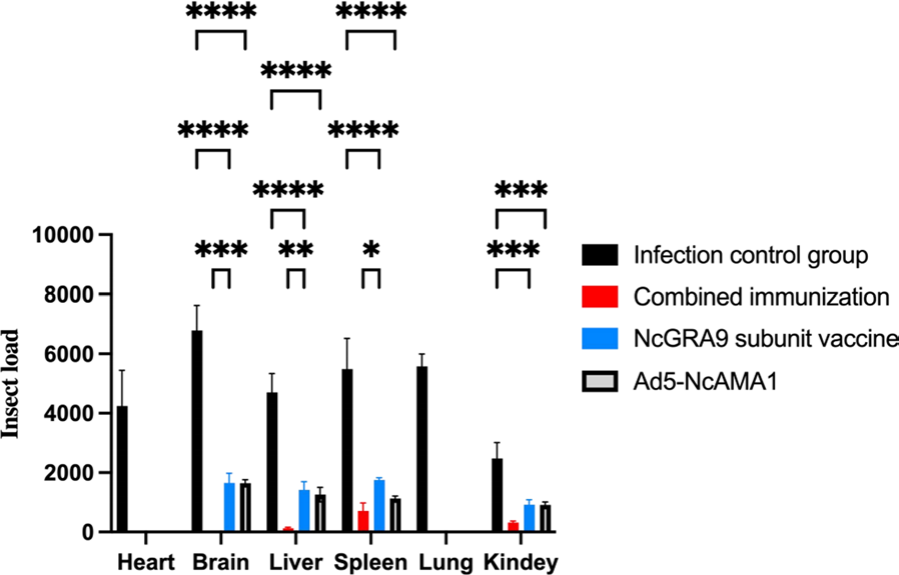
Absolute fluorescence quantitative qPCR results of various organs in mice. *P < 0.05, ** *P* < 0.01, *** *P* < 0.001, **** *P* < 0.0001

Fluorescence quantitative PCR showed that, after infection with *N. caninum*, the parasite load in the major organs in each immunized group of mice was significantly lower than that in the control group (*P* < 0.0001) (Fig. 5). The parasite load in the brain and liver in the NcGRA9 + Ad5-NcAMA1 group was lower than that in the NcGRA9 and Ad5-NcAMA1 groups (*P* < 0.05 and *P* < 0.0005). No *N. caninum* bodies were detected in the heart and lung tissues of the immunized groups.

### Detection of immunoglobulins in serum of immunized cattle

**Fig. 6:**
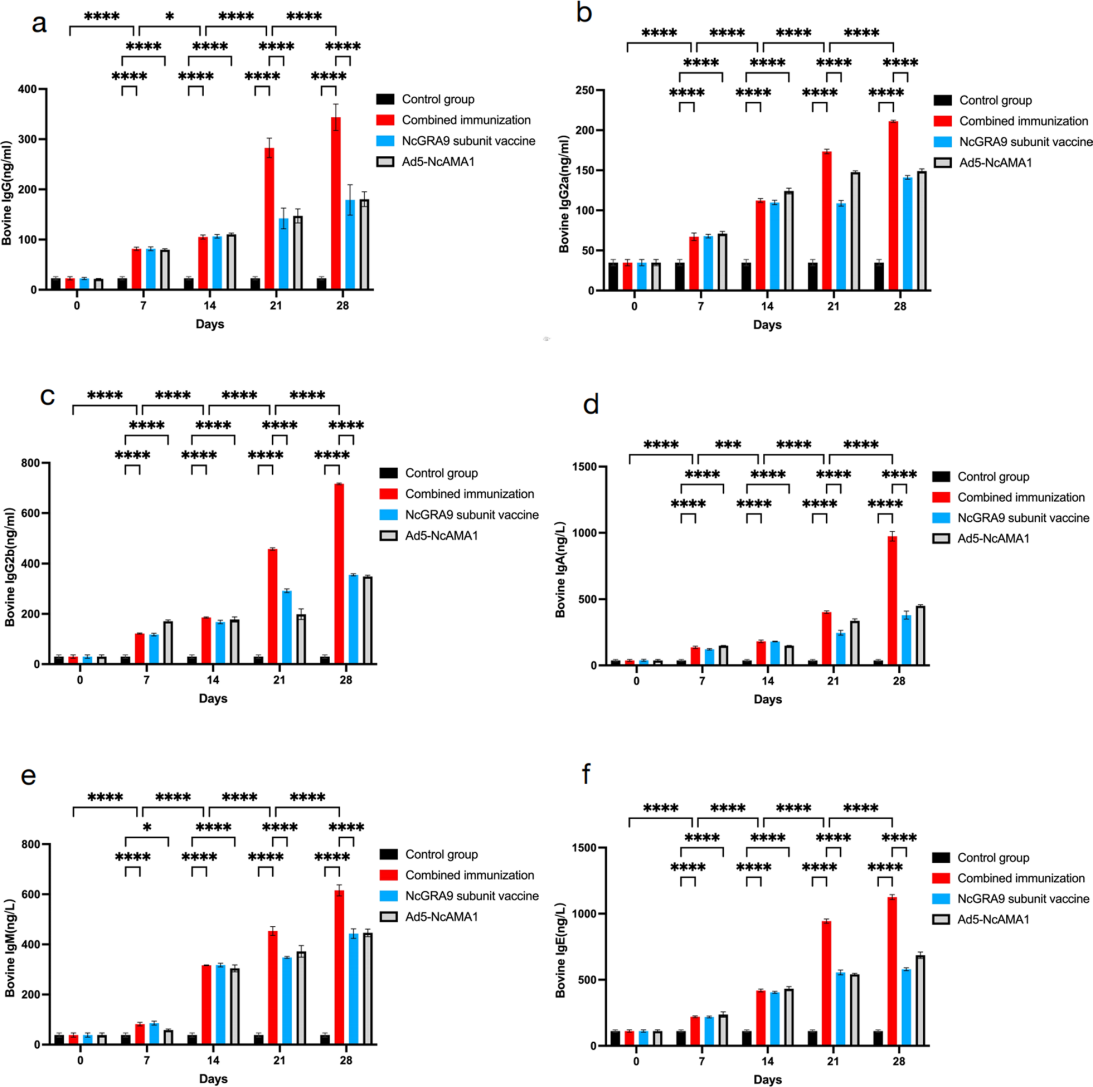
Serum immune antibody levels of bovines in different immune groups (combined immune group; NcGRA9 group; Ad5-NcAMA1 group). **a** ELISA showing the IgG level of bovines; **b** ELISA showing the IgG2a level of bovines; **c** ELISA showing the IgG2b level of bovines;**d** ELISA showing the IgA level of bovines. **e** ELISA showing the IgM level of bovines; **f** ELISA showing the IgE level of bovines. *P < 0.05, ** *P* < 0.01, *** *P* < 0.001, **** *P* < 0.0001

IgG IgG2a, IgG2b, IgA, IgM and IgE in bovine serum after immunization were measured by ELISA (Fig. 6); all increased significantly with the number of immunizations (*P* < 0.0001). At day 7 of immunization, the IgA index was significantly higher in the Ad5-NcAMA1 group than in the NcGRA9 group (*P* < 0.05). At day 14 of immunization, the IgA level was significantly higher in the NcGRA9 + Ad5-NcAMA1 and NcGRA9 groups than in the Ad5-NcAMA1 group (*P* < 0.05 and *P* < 0.001). IgG2a level was significantly higher in the Ad5-NcAMA1 group than in the other two groups at day 14 of immunization (*P* < 0.001). The level of IgG2b was significantly higher in the Ad5-NcAMA1 group than in the other two groups at day 7 of immunization (*P* < 0.0001). The level of IgM showed a significant decrease at day 7 of immunization (*P* < 0.05). The level of IgE was significantly higher in the Ad5-NcAMA1 group than in the NcGRA9 group at day 14 of immunization (*P* < 0.05). There was no significant difference between the Ad5-NcAMA1 and the NcGRA9 groups from day 7 to 28 of immunization. From day 21 to 28 of immunization, the levels of IgA, IgG2a, IgG2b, IgM, IgE and IgG in the combined immunization group were significantly higher than those in the NcGRA9 and Ad5-NcAMA1 groups (*P* < 0.0001).

### Detection of serum cytokine levels in immunized cattle

**Fig. 7:**
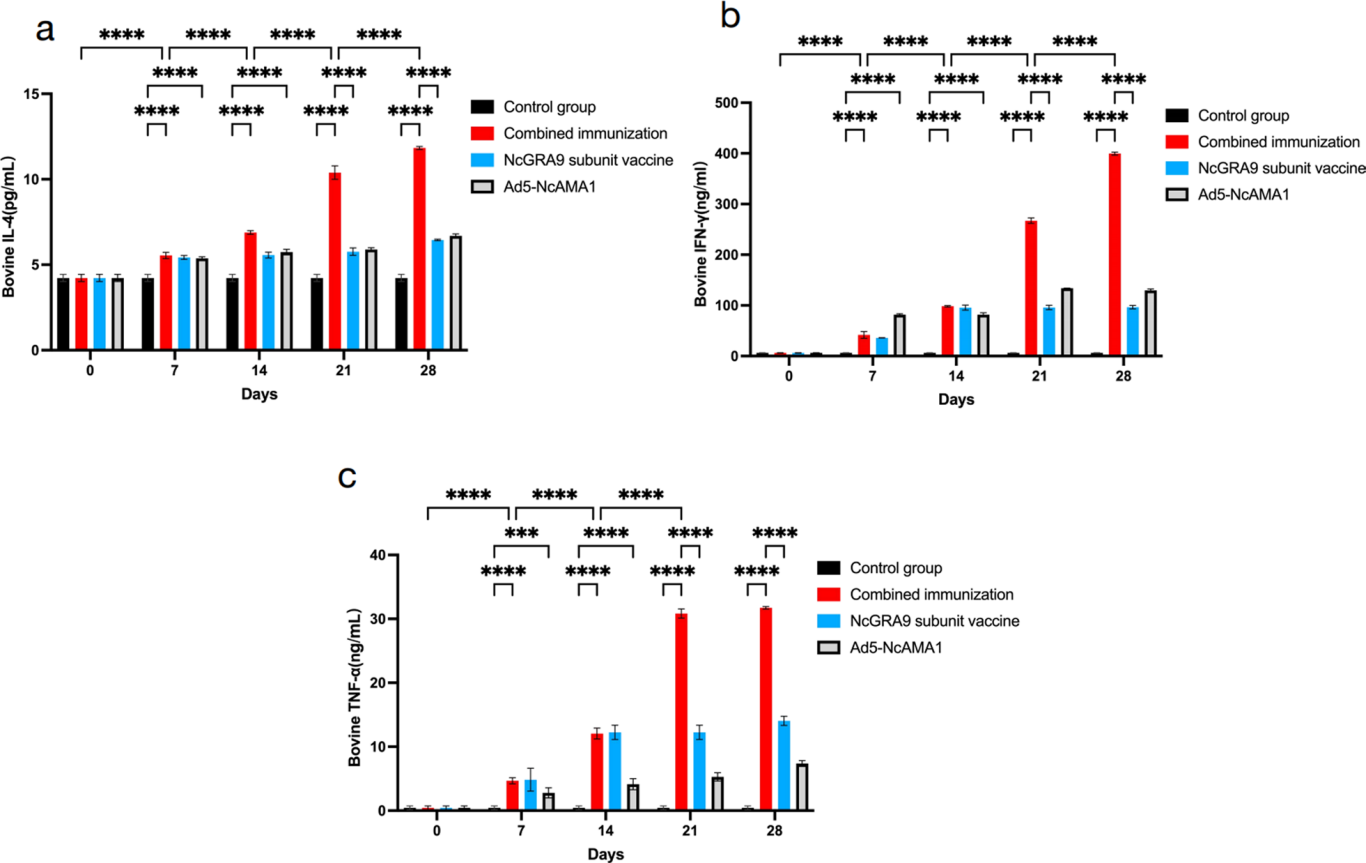
Cytokine antibody levels of bovines in different immune groups (combined immune group; NcGRA9 group; Ad5-NcAMA1 group). **a** ELISA showing the IL-4 level of bovines; **b** ELISA showing the IFN-γ level of bovines; **c** ELISA showing the TNF-α level of bovines. *P < 0.05, ** *P* < 0.01, *** *P* < 0.001, **** *P* < 0.0001

The expression of cytokines IL-4, IFN-γ and TNF-α in bovine sera were detected by ELISA (Fig. 7). The expression of IL-4, IFN-γ and TNF-α in each immunized group was significantly increased compared with expression on day 0 (*P* < 0.0001). Expression of IL-4 in the combined immunization group was significantly higher than that in the NcGRA9 and Ad5-NcAMA1 groups at days 14 and 28. In the Ad5-NcAMA1 group, expression of IFN-γ was significantly higher than in the other immunized groups on day 7 (*P* < 0.0001) but was significantly lower in the Ad5-NcAMA1 group on day 14 (*P* < 0.0001). TNF-α expression was significantly lower in the Ad5-NcAMA1 group on days 7 and 14 compared with the combined immunization group and the NcGRA9 group (*P* < 0.001). From day 21 to day 28 of immunization, TNF-α expression in the combined immunization group was significantly higher than in the other three groups (*P* < 0.0001).Expression of cytokines IL-4, IFN-γ and TNF-α on days 21 and 28 was significantly elevated in the combined immunization group compared with the Ad5-NcAMA1 and NcGRA9 groups (*P* < 0.0001).

### Sixteen tests for bovine health after immunization

Sera were extracted from bovine blood after collection and analyzed using a fully automated animal biochemical analyzer (Table 2). No abnormality was observed in the Ad5-NcAMA1 group; creatine kinase was slightly elevated in the combined immunization group and the NcGRA9 group; whole protein was slightly elevated in the NcGRA9 group. However, there was no significant difference compared with the normal control group, indicating that the vaccine had no effect on the liver and kidney functions of cattle.

**Table 2:**
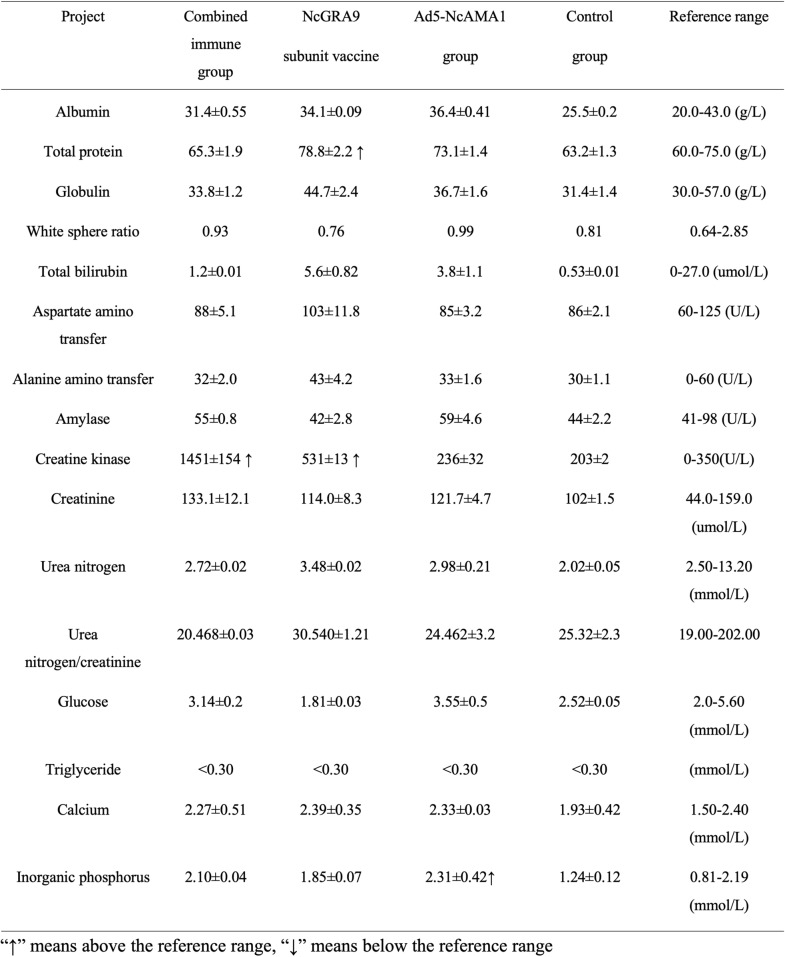
Sixteen health tests of bovines after immunization

## Discussion

The impact of neosporosis on the livestock industry is increasing every year, but there are no effective vaccines and drugs to prevent and control it [19–21]. Several *N. caninum* GRAs have been reported, among which NcGRA9 is less well studied. NcGRA9 is located on a single copy of the gene on chromosome XII of the *N. caninum* genome and contains two exons and one intron. The *N. caninum* GRA9 gene was found to be highly homologous to the *T. gondii* GRA9 gene, and the sequence comparison of NcGRA9 and TgGRA9 proteins showed 60% identity. Immunofluorescence studies have shown that NcGRA9 is present in a punctate pattern and distributed throughout the parasite [22]. This is a typical pattern for GRA proteins. After invasion of the host cell, NcGRA9 is secreted into the parasitophorous vacuole (PV); some is retained in the vesicular space near the tachyzoites, and some is located at the periphery of the PV. Secretion into the PV after invasion is characteristic of GRA proteins [23].

Jin et al. [24] expressed and characterized NcGRA2, demonstrated the effect of NcGRA2 on *N. caninum* immunity in mice and established an indirect ELISA method. Xing et al. [25] reported expression of the NcGRA6 gene and preliminary analysis of its immunization effect. The antibody titer of NcGRA6 protein reaches 10^6^–10^7^ after three immunizations, and the parasite load in the brain of the immunized mice was significantly reduced compared with that of the unimmunized group. Du et al. [26] conducted preliminary analyses to localize NcGRA16 and confirmed that it is a virulence invasion gene. They found that △NcGRA16 *N. caninum* had a greater ability and virulence to invade host cells after constructing the △NcGRA16 strain using CRISPR-cas9 technology. However, no relevant studies on NcGRA9 have been reported. In particular, the function of NcGRA9 and the role of this protein in the life cycle of *N. caninum* are unknown. To understand the expression of NcGRA9 gene and its immunogenicity as a candidate vaccine antigen, we cloned and expressed the NcGRA9 gene in this study. A recombinant protein of 45.1 kDa was obtained with good reactivity, and a subunit vaccine was prepared for inoculation of C57BL/6 mice, which achieved good results.

The AMA1 gene is homologous in all apicomplexan parasites. AMA1 protein is also expressed in *N. caninum* and *T. gondii* during the bradyzoite and tachyzoite phases. AMA1 proteins are typical single-copy transmembrane proteins [14, 15]. AMA1 recombinant protein can inhibit *N. caninum* invasion of the host [14], and amplified expression of the AMA1 gene has shown that the gene inhibits the invasion of *N. caninum* [27–29]. Among the three structural domains of AMA1, DI and DII play an obvious inhibitory role, and DII is also able to stimulate a high level of cellular immunity and humoral immune response [30, 31]. Therefore, AMAI gene is a promising candidate vaccine antigen.

Subunit vaccines have become a hotspot for vaccine development in recent years due to their advantages of better safety, good immunological effects and affordability [32]. *Neospora caninum* is an intracellular parasite. Studies have shown that IgG1, IgG2a, IL-4 and IFN-γ play an important role in the immune response to *N. caninum* [17]. IgG antibody plays an important role in immunity against *N. caninum*: IgG1 is involved in removal of the parasite, and IgG2 is related to control of abortion. After infection, T cells are activated; of these, CD4^+^ and CD8^+^ cells play an important role in host immunity against *N. caninum*, with CD4^+^ cells being more effective than CD8^+^ cells. Th1 and Th2 CD4^+^ cells secrete a variety of cytokines, such as IL-1, IFN-α, IL-2 and IFN-γ, which can enhance resistance to *N. caninum* [33, 34]. In particular, IFN-γ can regulate *N. caninum* proliferation to a large extent, and IFN-γ deficiency can make the host susceptible to *N. caninum*.

The development of vaccines against adenoviruses is very promising [35]. Adenoviruses, as a multifaceted structure without capsid, have been constantly and rapidly developing after being applied in clinical trials, in which the full sequences of the Ad2 and Ad5 types are the most comprehensively understood [36, 37]. Adenoviruses have virtually no adverse effects except for the possibility that they may cause very minor symptoms and adenoviral vectors are in a free state outside the genome of the host cell. Recombinant adenoviral vector vaccines consistently produce potent antibodies and enhance immunity in animals that have their own maternal antibodies [38–40]. Guo et al. [41, 42] used the transpose-Ad(TM) adenoviral vector system to construct a recombinant Ad5-NcAMA1 vaccine against *N. caninum*, which was used to vaccinate BALB/c mice using a prime-boost strategy. Ad5-NcAMA1 induced humoral and cellular immune responses. In the present study, NcGRA9 subunit vaccine, Ad5-NcAMA1 adenovirus recombinant vaccine and NcGRA9 + Ad5-NcAMA1 combination vaccine were used to immunize BALB/c mice. Cellular and humoral immune responses were stimulated in the immunized mice, and all the immunological parameters were significantly improved after six immunizations. The immune responses were greater after immunization with the NcGRA9 + Ad5-NcAMA1 vaccine. Two weeks after immunization, biochemical analysis of whole blood showed that the vaccine had no effect on liver and kidney functions. The vaccines reduced mortality after parasite challenge. Survival rate was 70%with NcGRA9 vaccine, 75% with Ad5-NcAMA1 and 85% with NcGRA9 + Ad5-NcAMA1 compared with only 10% in the unvaccinated control group. The parasite load in brain, liver and spleen tissues was detected by fluorescence quantitative PCR and was significantly lower in the immunized mice. The NcGRA9 + Ad5-NcAMA1 group had the lowest parasite load. These results demonstrated that the vaccines had a protective effect against *N. caninum* infection in mice.

IgG, IgA, IgM, IgE and subclasses IgG2a and IgG2b are important immune indicators in bovine sera [43–45], with IgM and IgE closely related to parasitic infections [46]. IL-4, IFN-γ and TNF-α are important for Th1/Th2 balance in bovines [47–49]. Similarly, IL-4 and IFN-γ play important roles in parasite clearance. Based on our studies with C57BL/6 mice, we also immunized Yanbian yellow cattle with the same antigenic vaccines. Similar to Chacón-Díaz et al. [50], we found a gradual increase in the various immune factors in the serum of cattle immunized with NcGRA9 and the Ad5-NcAMA1 vaccines, but the rate of increase slows down after days 21–28 of immunization. This situation was resolved by the NcGRA9 + Ad5-NcAMA1 vaccine, which showed an increase in all the immune factors compared with either vaccine alone. The same trend was observed for IL-4, IFN-γ and TNF-α. The increase in IgM, IgE, IL-4 and IFN-γ indicated that all three vaccines had good immunogenicity against *N. caninum*. Similar to mice, it was found that immunization did not affect the liver and kidney functions of cattle.

## Conclusions

In this study, NcGRA9 recombinant subunit vaccine and Ad5-NcAMA1 recombinant adenoviral vector vaccine were prepared. The two vaccines were used to immunize C57BL/6 mice and Yanbian yellow cattle individually and in combination. The vaccines induced high levels of humoral and cellular immune responses in mice, significantly reduced the parasite load in brain and liver tissues and had good immunoprotective effects. The vaccines also induced a high level of humoral and cellular immune response in cattle. Biochemical tests showed that the vaccines had no effect on liver and kidney function in mice and cattle and that the combined vaccine had the best effect. In conclusion, NcGRA9 + Ad5-NcAMA1 may be a candidate vaccine for bovine neosporosis.

## Data Availability

No datasets were generated or analysed during the current study.
